# Intravitreal melphalan for persistent retinoblastoma vitreous seeds

**DOI:** 10.3205/oc000190

**Published:** 2022-02-08

**Authors:** Marie Jeazelle H. Redondo-Villanueva, Gary John V. Mercado

**Affiliations:** 1 University of the Philippines Manila, Philippine General Hospital, Department of Ophthalmology and Visual Sciences, Manila, Philippines

## Abstract

A 7-month-old male presented with bilateral retinoblastoma, initially group E OD and group C OS (International Intraocular Retinoblastoma Classification). The patient underwent enucleation with adjuvant chemotherapy and radiotherapy on the right eye for extraocular retinoblastoma. The main tumors on the left eye regressed with combined neoadjuvant chemotherapy with focal therapy, but had persistent sphere vitreous seeds overlying the tumor at the superotemporal mid-periphery and at the inferior periphery. Intravitreal injection of melphalan was performed. Vitreous seeds were clinically undetectable after 2 cycles of injection. Six months from the 2^nd^ intravitreal injection of melphalan, there was no evidence of new tumors nor vitreous seeds in the left eye. No documented complications of intravitreal melphalan injection were experienced. This is the first documented successful treatment of vitreous seeds with intravitreal melphalan at the Ocular Oncology/Retinoblastoma Unit of the University of the Philippines, Philippine General Hospital.

## Background

Vitreous seeding is one of the most challenging features to control in intraocular retinoblastoma management. It results from tumor cells migrating into the vitreous, frequently depositing and growing on the retina [1]. Munier [2] classified 3 different morphologic types of vitreous seeds. They found that aside from differences in morphologic features, these classes of seeds also vary in their response to treatment [3].

Before the pioneering work of Kaneko and Suzuki in the 1990s, the only globe-sparing options available for persistent vitreous seeds were external beam radiotherapy (EBR), and brachytherapy. Primary intravenous systemic chemotherapy provided limited outcomes for vitreous seed control [1]. In fact, only 27–47% of the eyes did not require enucleation and/or EBR with primary systemic chemotherapy alone [4]. With the advent of local chemotherapy (intravitreal or ophthalmic artery injection), outcomes of group D eyes significantly improved with 87% to 100% vitreous seed control documented in several centers using this technique [4], [5], [6], [7], [8].

We present a case of bilateral retinoblastoma with persistent vitreous seeds, despite systemic intravenous chemotherapy and successfully treated with intravitreal injection of melphalan. It aims to outline management options for recalcitrant vitreous tumor seeding. 

## Case description

A 7-month-old male presented with buphthalmos of the right eye due to group E retinoblastoma. On examination, the patient had no dazzle on the right eye, but fixes and follows with the left. The right eye had a shallow anterior chamber and a horizontal corneal diameter of 13 mm with no view on indirect ophthalmoscopy. B-scan ultrasound of the right eye revealed an intraocular mass consistent with retinoblastoma. CT scan showed no evidence of extrascleral, intracranial or optic nerve invasion. Dilated fundoscopy of the left eye revealed 3 retinal tumors measuring 3.5x3.5x3 mm, 9x9x8 mm with overlying vitreous seeds, and 5x5x4.5 mm with surrounding vitreous seeds extending up to mid-vitreous. The patient was diagnosed to have bilateral retinoblastoma, IIRC group E OD and group C OS.

Enucleation of the right eye was performed and histopathology was read as extraocular retinoblastoma with high risk features (massive choroidal invasion but no scleral invasion, post-laminar optic nerve invasion) with optic nerve cut end positive for tumor invasion. Adjuvant intravenous systemic chemotherapy (vincristine, etoposide, carboplatin) and orbital radiotherapy was performed.

**Figure 1:**
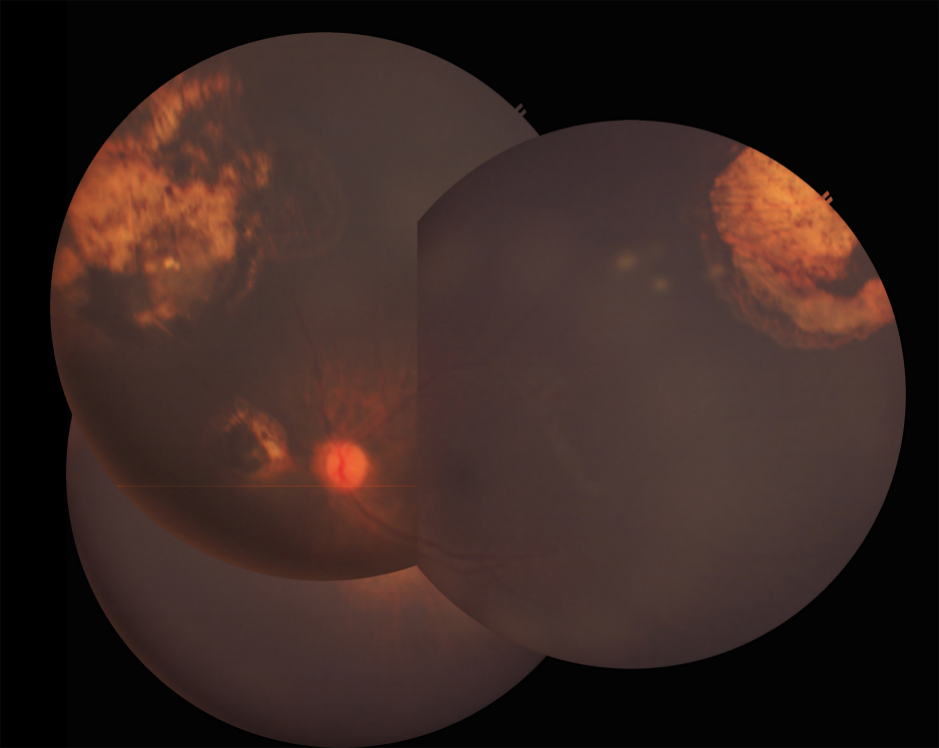
Fundus photo montage showing persistent sphere vitreous seeds overlying the tumor superotemporally and at the inferior periphery of the left eye

Combined intravenous chemotherapy with focal therapy (thermotherapy and cryotherapy) resulted in regression of the retinal tumors of the left. Partial response of the vitreous seeds was seen, but sphere seeds persisted over the superotemporal tumor and at the inferior periphery (Figure 1 (Fig. 1)). Intravitreal injection of melphalan 20 µg/0.05 cc was performed at the inferotemporal midperiphery (4 o’clock) with cryotherapy (triple freeze-thaw) of the injection site, following the procedure described by Shields et al. [8]. The patient received 2 cycles of injection 6 weeks apart, the first concurrent with the 6^th^ cycle of systemic chemotherapy, and the next 6 weeks thereafter.

**Figure 2:**
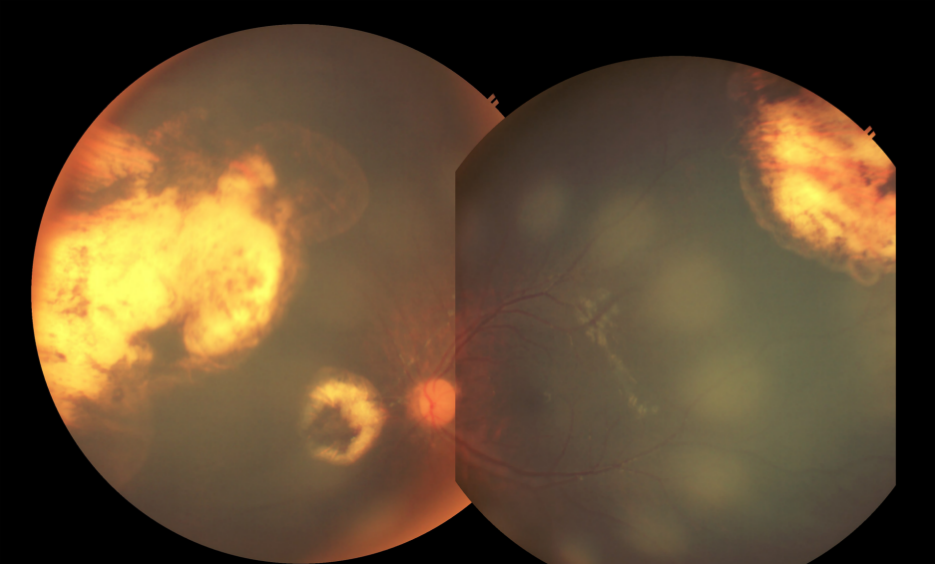
Fundus photo montage showing clinically undetectable (regression type 0) vitreous seeds 6 months after 2 cycles of intravitreal melphalan injection

Recurrent vitreous seeds overlying the tumor at the superotemporal mid periphery and at the inferior periphery were clinically undetectable (regression type 0) after 2 cycles of intravitreal melphalan injection (Figure 2 (Fig. 2)). There were no new tumors and vitreous seeding observed on the left eye 6 months from the last intravitreal injection of melphalan. There were no documented complications of intravitreal melphalan injection.

## Discussion

Up until recently, vitreous seeding has been one of the major limiting factors for globe salvage in advanced intraocular retinoblastoma. Recent studies involving intravitreal chemotherapy have been found to be safe and efficacious in controlling vitreous seeding [1], [4], [5], [6], [7], [8], [9]. In a review, Kaneko and Suzuki reported a 51.3% long-term rate of eye preservation with intravitreal injection of melphalan. The study has revolutionized the treatment for retinoblastoma because it showed that intravitreal chemotherapy is not as dangerous or toxic as previously feared [1]. Refinement in the injection technique as well as dose adjustment have made it even safer and more efficient. In 2012, Munier et al. reported an 83% success rate of vitreous seed control defined as absence of vitreous and/or epiretinal relapse and absence of the need to enucleation and/or EBR [4]. In the report, they described their needle tract sterilization and safety precaution techniques [4].

Munier introduced a classification scheme for vitreous seeds in retinoblastoma based on differences in their morphologic features as dust, sphere, and clouds [2]. Francis et al. found that each seed type has a significantly distinct time to regression, number of injections, and cumulative and mean dose of melphalan needed for seed control. Dusts were the fastest to regress, needing the least number of injections and lowest dose of melphalan, whereas clouds were the opposite. They concluded that vitreous seed classification seems to be predictive of the time it will take to control the vitreous seeds, and the number of injections and dose of melphalan necessary. Overall, they reported a 2-year Kaplan Meier estimate for ocular survival at 90.4% and patient survival of 100% [3].

Optimal dosage for intravitreal melphalan injection was also investigated. In 2012, Ghassemi and Shields used low dose (8 µg/0.1 mL) intravitreous melphalan for vitreous seeding wherein they documented rapid seed regression and minimal side effects, but later noted vitreous seed recurrence. A 50-µg dose melphalan for 4 cases provided an immediate, short-term, and long-term control of 100%. However, such dose was found to have complications of cataract, vitreous hemorrhage, subretinal hemorrhage, severe hypotonia, and phthisis, leading to enucleation in 2 cases. The authors suggested that an intermediate dose of 20 µg to 30 µg/0.1 mL melphalan was ideal for tumor control and globe safety, avoiding recurrences seen with lower doses and toxicities observed with higher doses [5]. Shields et al. reported 100% vitreous seed control with little complication in a preliminary study using a standard dose of 20 µg to 30 µg/0.1 mL melphalan with a median number of 6 injections [6]. This was further refined when Shields et al. presented their retrospective interventional case series of 40 eyes with viable vitreous seeding, wherein they concluded that intravitreal melphalan and/or topotecan injection provides lasting tumor control at 3 years with approximately 4 injections for melphalan and 3 injections for topotecan. The most common complications reported were retinal pigment epithelial mottling (32% risk per eye), paraxial subclinical cataract (25%), minor transient vitreous hemorrhage (13%), localized retinal hemorrhage (5%), and corneal epitheliopathy (5%). There was no case of endophthalmitis, extraocular tumor extension, metastasis, or death reported after 192 consecutive injections [8].

This report describes a case of recalcitrant retinoblastoma vitreous seeds despite intravenous systemic chemotherapy, which was successfully controlled with intravitreal melphalan. Although it is currently used in specialized retinoblastoma care centers in the world, intravitreal injection of chemotherapy has just become available in the Philippines. This is the first documented successful retinoblastoma vitreous seed control utilizing intravitreal melphalan in the University of the Philippines, Philippine General Hospital.

## Conclusion

This is the first documented successful retinoblastoma vitreous seed control utilizing intravitreal melphalan injection in the University of the Philippines, Philippine General Hospital. Intravitreal injection of melphalan was found to be safe and efficacious in control of vitreous seeds and eye preservation in retinoblastoma. Optimal dosage and proper injection technique must be used to prevent potential complications.

## Notes

### Competing interests

The authors declare that they have no competing interests.
